# Editorial: Arrhythmogenic Substrates in Diabetes and Obesity

**DOI:** 10.3389/fphys.2019.00549

**Published:** 2019-05-10

**Authors:** John P. Morrow, Fadi G. Akar, Ademuyiwa S. Aromolaran

**Affiliations:** ^1^Division of Cardiology, Department of Medicine, Vagelos College of Physicians and Surgeons, Columbia University, New York, NY, United States; ^2^Cardiovascular Research Center, Icahn School of Medicine at Mount Sinai, New York, NY, United States; ^3^Cardiovascular Research Program, VA New York Harbor Healthcare System, Brooklyn, New York, NY, United States; ^4^Department of Cell Biology, State University of New York Downstate Medical Center, Brooklyn, New York, NY, United States

**Keywords:** arrhythmia, sudden cardiac death, diabetes, obesity, lipotoxicity, mitochondria

The increasing prevalence of obesity and type 2 diabetes mellitus (T2DM) is a critical public health concern. This is due, in part, to the associated increased incidence of fatal arrhythmias that cause sudden cardiac death (SCD) (Ackerman et al., 2011). Recently, estimation by the NIH shows that obesity and related comorbidities impact about 17% of children and young adults in the United States, while more than 2/3 of adults are either overweight or obese (Jensen et al., 2014). Chronic consumption of calorically rich and fatty diets is a major contributor to the obesity/T2DM epidemic (Virtanen et al., 2012). As such, efforts to understand the functional relationships between existing and newly defined dietary-activated molecular signaling pathways are an essential part of the effort to understand the pathogenesis of arrhythmias (ventricular and supraventricular). This objective can be advanced by understanding the fundamental mechanisms that underlie adverse structural and electrical remodeling, impaired mitochondrial metabolism, lipotoxicity, and inflammation in obese/diabetic hearts. Consequently, identifying whether a small set of unifying mechanisms underlie metabolic disorders could help development of novel therapeutic targets for arrhythmias in patients. In this Research Topic we introduce a series of articles written by authors with expertise in various aspects of metabolic regulatory pathways in animal studies and how this relates to translational outcomes in obese/T2DM patients. We believe that the ideas presented in these articles can enhance our knowledge of the pathophysiology of metabolism and cardiovascular diseases in general.

The research topic opens with the review article by Lubbers et al. which focuses on the pathological link between obesity and atrial fibrillation (AF), the most common arrythmia affecting both men and women, and associated with increased morbidity and mortality (Abed and Wittert, 2013). To date the mechanisms that link obesity and increased susceptibility to AF is poorly understood. The authors highlight the relevance of potential crosstalk and coupling pathways that may be involved in adverse atrial structural and electrical remodeling. Further, the consequences of obesity-mediated altered systemic inflammatory signaling and redox regulation on structural (fibrosis and atrial size), and electrical (ion channels and conduction pathways) integrity in the heart are emphasized. In addition, because congenital mutations in major atrial ionic channels underlie some cases of AF (Ackerman et al., 2011), they raise the possibility of how and whether obesity mechanisms may modulate penetrance of associated ion channel AF variants. The authors provide detailed discussions of dietary and therapeutic interventions for AF.

The review by Alí et al. discusses excessive accumulation of saturated free-fatty acids (or lipotoxicity) as a key mechanism underlying arrhythmogenic events in obesity-mediated arrhythmias. Ventricular and atrial remodeling in lipotoxicity, a disease that is associated with macrophage infiltration, is linked to activation of toll-like receptors, pathological increases in pro-inflammatory cytokines and adverse ion channel remodeling. The authors' conclusion supports the notion that future studies are needed to fully understand the molecular mechanisms of downstream inflammatory pathways that are activated in both macrophages and cardiomyocytes in obesity.

The article by Pabon et al. highlights the effects of adipocyte molecular mechanisms on adverse atrial and ventricular electrical activity resulting in atrial fibrillation and ventricular arrhythmias, with a focus on adipocyte location and the spatial secretion of pathological levels of adipokines and metabolites and free-fatty acid accumulation. The authors state that local and remote adiposity may control activation of distinct downstream metabolic pathways with a particular emphasis on pathways that initiate cardiomyocyte cell death.

It is widely known that diabetic cardiomyopathy contributes to fatal arrhythmias and sudden cardiac death in patients (Eranti et al., 2016), and two reviews on this topic provide molecular insights regarding the role of defective mitochondrial function in the pathogenesis of arrhythmias. First IIkan and Akar provide a detailed review of a unique role of the mitochondrial translocator protein (TSPO) in the link between impaired mitochondrial stability and arrhythmias. The review by Song et al. provides an elegant discussion of how fibrosis, impaired function of calcium handling proteins, dysfunction of ion channels and intracellular calcium release channels may contribute to mitochondrial dysfunction in diabetic hearts. The authors conclude that if we fully understand the function of individual or multiple combinations of these mitochondrial downstream pathways, we may be able to provide mechanism-based insights that will inform rational development of novel therapies for diabetic cardiomyopathies.

The review by Hamilton and Terentyev also aims to shed light into the notable links between major cardiomyocyte calcium channels, calcium handling proteins, and diabetes-associated cardiomyopathy and arrhythmogenesis. The review provides an elegant insight into the functional interplay between ventricular arrythmias, heart failure and SCD with focused discussions on molecular processes of dysregulated local calcium release and its molecular partners. The authors conclude with descriptions of how therapeutic regulation of triggered Ca molecular processes could be beneficial to patients with obesity-related arrhythmogenesis.

Metabolic syndrome (Obesity, T2DM, hyperglycemia, and dyslipidemia) remains one of the major public health challenges including being a risk factor for the onset of arrhythmias in patients worldwide. Thus, there is a global unmet and urgent medical need to develop safe and effective treatment strategies. The review by Homan et al. provides a detailed and rigorous clinical perspective into future research directions and challenges associated with prediction of arrhythmogenesis and vulnerability to SCD in patients.

Overall, this research topic highlights important and unanswered scientific questions with direct implications for human disease. The good news is that there is considerable room for improvement in developing new therapies to manage cardiomyopathies and arrhythmogenesis in patients with metabolic disorders. The continuing discovery of new technologies and interventions offer a distinct promise that future collaborative efforts related to the theme of the research topic, are likely to significantly improve existing knowledge of life-threatening arrhythmias caused by obesity and T2DM.

## Author Contributions

All authors listed have made a substantial, direct and intellectual contribution to the work, and approved it for publication.

### Conflict of Interest Statement

The authors declare that the research was conducted in the absence of any commercial or financial relationships that could be construed as a potential conflict of interest.
